# Series of Norovirus Outbreaks Caused by Consumption of Green Coral Lettuce, Denmark, April 2016

**DOI:** 10.1371/currents.outbreaks.115761d5d6de6a8bc7dd4b41f0f5f142

**Published:** 2016-10-04

**Authors:** Luise Müller, Lasse Dam Rasmussen, Tenna Jensen, Anna Charlotte Schultz, Charlotte Kjelsø, Celine Barnadas, Kim Sigsgaard, Anne Ribert Larsen, Carl Widstrup Jensen, Simon Jeppesen, Katrine Uhrbrand, Nikolas Hove, Kåre Mølbak, Steen Ethelberg

**Affiliations:** Department of Infectious Disease Epidemiology, Statens Serum Institut, Copenhagen, Denmark; Microbiological Diagnostics and Virology, Statens Serum Institut, Copenhagen, Denmark; Division of Food and Feed Safety, Danish Veterinary and Food Administration, Ministry of Environment and Food, Glostrup, Denmark; Division of Microbiology and Production, National Food Institute, Technical University of Denmark, Søborg, Denmark; Department of Infectious Disease Epidemiology, Statens Serum Institut, Copenhagen, Denmark; European Programme in Public Health Microbiology, European Centre for Disease Prevention and Control, Stockholm, Sweden; Department of Microbiological Diagnostics & Virology, Statens Serum Institut, Copenhagen, Denmark; Food Control Office Copenhagen, Danish Veterinary and Food Administration, Copenhagen, Denmark; Food Control Office Zealand/Funen, Danish Veterinary and Food Administration, DenmarkFood Control Office Zealand/Funen, The Danish Veterinary and Food Administration, Denmark; Food Control Office Copenhagen, Danish Veterinary and Food Administration, Copenhagen, Denmark; Food Control Office Copenhagen, Danish Veterinary and Food Administration, Copenhagen, Denmark; Division of Microbiology and Production, National Food Institute, Technical University of Denmark, Søborg, Denmark; Division of Food and Feed Safety, Danish Veterinary and Food Administration, Ministry of Environment and Food, Glostrup, Denmark; Department of Infectious Disease Epidemiology, Statens Serum Institut, Copenhagen, Denmark; Department of Infectious Disease Epidemiology, Statens Serum Institut, Copenhagen, Denmark

## Abstract

Introduction: In early April 2016, an unusual high number of point-source outbreaks of gastrointestinal disease were reported to occur in Denmark.

Methods: Outbreaks were individually investigated. Two analytical studies were performed. Patient stool samples collected and analysed; positive stool samples were sequenced over the polymerase and/or capsid gene areas. Implicated lettuce heads were collected and analysed for the presence of norovirus. Foods were traced-back and traced-forward and international alert systems applied.

Results: A total of 23 linked point-source outbreaks occurred over the course of one week. Fresh green coral lettuce (Lollo Bionda lettuce) had been consumed in all settings. In a cohort study including 234 participants a dish containing green lettuce was associated with illness. Norovirus of Genogroup I (GI) was detected in samples from 28 patients comprising eight of the outbreaks. Sequencing showed GI.P2-GI.2. GI norovirus was detected in one of 20 examined lettuce heads. All lettuce consumed was supplied by the same packer who in turn had bought the lettuce from a wholesaler in France. The two lots of lettuce came from two different growers in different parts of France.

Discussion: Green coral lettuce produced in France was found to have caused a large series of linked norovirus outbreaks in Denmark as established by a number of lines of evidence. A similar incidence occurred in 2010. Fresh lettuce increasingly appear to be a risk food for norovirus infections.

## INTRODUCTION

In Denmark, as indeed in most countries, norovirus infections are not covered by surveillance. However, they frequently cause outbreaks in health care institutions and other closed settings, and food is often the vehicle of infection. In Denmark, typically 60-100 foodborne outbreaks are registered annually. Of these, norovirus is found to be the etiological agent in between 35%-50%, thus making it the single most frequent cause of foodborne outbreaks.^1^^,^ 2 ^,^ 3 Most outbreaks occur because the food is contaminated during preparation and serving; a recent review of 191 norovirus outbreaks in Denmark found that 27% of the outbreaks were caused by contamination in the production chain.^3^ Such contaminated food items involved have primarily been frozen raspberries, oysters and drinking water.^4^^,^ 5

In the first week of April 2016, a higher than usual number of point-source outbreaks of gastroenteritis were reported to the food authorities throughout the country. The Food Control Office in Zealand/Funen reported 5 April on initial results from concurrent investigations of five outbreaks related to cafés or restaurants. A common link, possibly green coral lettuce (type: *Lollo Bionda*), was suspected. This led to the initiation of a national outbreak investigation with the aim to assess the magnitude of this public health event, to establish if a link between the outbreaks existed, to determine the causative agent and reveal the source in order to stop the outbreaks. We here report on the results of these investigations.

## METHODS

The outbreak investigation was coordinated by the Danish Veterinary and Food Administration (DVFA) in cooperation with Statens Serum Institut (SSI) and The National Food Institute at the Technical University of Denmark (DTU-FOOD). The Danish Regional Food Control Offices, under the DVFA, conducted separate local outbreak investigations. An outbreak was considered a part of the meta-outbreak if the outbreak occurred in Denmark in March-April 2016 after consumption of green coral lettuce from France.

Local outbreak investigations were conducted via contact to outbreak venues and kitchens responsible for the catering to establish the circumstances of the outbreaks. Further investigations were then done to establish attack rate and food exposure details. Stool sample kits were distributed directly to patients by the local investigators where possible. Patients were encouraged to send in two separate stool samples to the SSI. Stool samples analysis was performed at the SSI and food sample analysis at DTU-FOOD. Unopened packages of green coral lettuce was collected from both kitchen and wholesale level if available. Twenty samples from eight different sites were available.

Two retrospective analytical cohort studies were conducted. Both used electronic questionnaires with links emailed to each of the cohort members and addressing details concerning symptoms and food exposures. The first study was initiated 13 April among participants in a one-day company seminar. It was primarily performed in order to obtain information on disease symptoms at a time, where the infectious agent was not known. This setting was chosen since it was a well-defined closed cohort and participants were easy to reach. Invitations to participate in a second study were sent out on 14 April to students and staff of a high school, where it was unclear from the beginning whether this outbreak was related to the other outbreaks. The objective of this investigation was to establish a possible food source of the outbreak. For the cohort studies, a case was defined as a person attending the common event and developing vomiting and/or diarrhea within three days.

Upstream tracing of lettuce from all outbreak settings was conducted by way of collecting and comparing purchase and delivery information in the period of one week prior to the outbreaks. Tracing was performed through the distribution chain to the packer in Denmark and from there to the wholesaler in France. This was then followed by forward tracing of the incriminated batches of lettuce, revealing the full distribution chain. Two EU communication networks were used, the Rapid Alert System for Food and Feed (RASFF) and the European Center for Disease Prevention and Control (ECDC) communication platform for potentially cross-border foodborne outbreaks (EPIS-FWD).^6^

Culturing was performed for pathogenic bacteria, including diarrhoeagenic *E. coli*, *Campylobacter*, *Salmonella* and *Yersinia.* 7 The presence of gastrointestinal viruses was assessed according to previous publications using a multiplex real-time PCR for rotavirus,^8^ sapovirus,^9^ human astrovirus and adenovirus (40+41).^10^ Norovirus analysis was performed using genogroup (G) specific primers^11^ by real-time RT-PCR. Sequencing of polymerase and/or capsid fragments obtained by RT-PCR using standard typing primers for the specific G, in this case GI^11^^,^ 12 ^,^ 13 ^,^ 14 was performed on an ABI 3500 (ThermoFisher scientific). Genotyping was performed both for sequences from patients and lettuce using the web-based norovirus typing tool NoroNet.^15^

Analyses for norovirus in lettuce were performed according to the ISO/DIS 15216-1 for quantitative determination of viruses in leafs^11^ with minor modifications. These included the use of 80 ml tris-glycine-beef-extract (1.5%) buffer and pectinase from *A. aculeatus* (3,800 units; Sigma-Aldrich, Brøndby, Denmark). Moreover, a chloroform-butanol extraction step of the virus-containing pellet was included. Plant RNA Isolation Aid (200 µl; Ambion, Naerum, Denmark) was added to the lysis step during nucleic acid extraction and BSA (1.25 µg; Invitrogen, Taastrup, Denmark) was added in the RT-PCR reaction mixture.^16^ Quality controls and criteria to assess the recovery efficiencies of viral genomic RNA^17^ and purity of RNA extracts^4^^,^ 18 were applied. Sequencing of capsid GI fragments obtained by RT-boosted-PCR using primers for GI^19^ was performed in both directions at Macrogen (Macrogen Inc., Korea).

## RESULTS

We identified 23 separate point-source outbreaks comprising 1497 exposed persons. A total of 412 (28%) persons were reported ill with diarrhea and/or vomiting (Table 1). Exposure occurred during a single week (31 March to 7 April 2016, Figure 1). Outbreaks occurred in several parts of the country (Figure 2), however 70% of outbreaks occurred in two of the five Danish administrative Regions, the Capital and Zealand Regions. The exposed individuals were part of groups being served food containing raw green lettuce, often as part of freshly made sandwiches. The attack rates in the individual outbreaks ranged from 5% to 100%.

**Figure:**
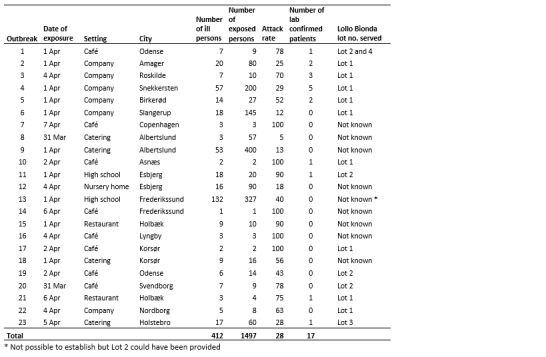
**Table 1.** Overview of individual outbreaks of norovirus infections related to consumption of green coral lettuce, Denmark, April 2016.

**Figure 1:**
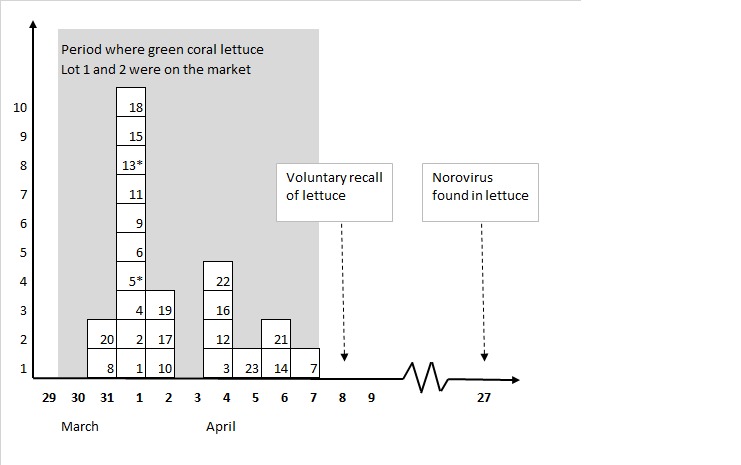
**Figure 1.** Timeline showing date of exposure for outbreaks of norovirus infections related to green coral lettuce, Denmark, April 2016 (numbers refer to the outbreak number shown in Table 1 and stars mark the two outbreaks for which cohort studies were conducted).

**Figure 2:**
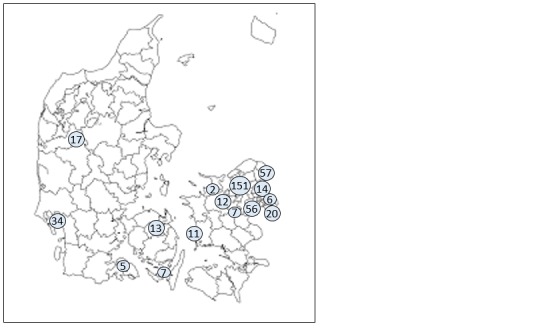
**Figure 2.** Map of Denmark showing the location of norovirus outbreaks related to green coral lettuce, April 2016. The map shows the outline of Denmark with its 98 Danish municipalities and the occurrence of outbreaks depicted as circles. The numbers indicate the combined number of patients within the area (n=412). Each circle may refer to more than one outbreak.

**Analytical investigations**

Cohort Study 1, performed in a company (outbreak 5, Table 1), showed disease onset and symptomatology to be in accordance with a point-source norovirus outbreak. The attack rate was 59%. All attendees had consumed sandwiches of which three types were served, however all containing the same type of lettuce. Responses from Cohort Study 2, performed among high school staff and students (outbreak 13, Table 1) showed that 250 out of 327 respondents had attended a school gala dinner on 1 April. Analysis was restricted to 234 individuals who took part in the dinner and party. Of these, 83 (35.5%) fulfilled the case definition. One food item was associated with an increased risk of illness; individuals who consumed the first course of the dinner (a starter consisting of salmon mousse on leafy greens) were seven times more likely to become ill (Relative Risk: 7.7; 95% confidence interval: 2.2-27). The chef initially stated that lettuce used in the kitchen was of Spanish origin and further that the starter had not contained any green coral lettuce. However, comments in the questionnaire from two students and a mobile phone photo taken during the party showed that salmon mousse was served on a bed of green coral lettuce. Further inquiries to the kitchen staff revealed that about 150 salmon starters served at the dinner contained green coral lettuce and further investigation at the wholesaler showed that the lettuce received and served was in fact of French origin, although it was not possible to establish the exact lot number.

**Trace-back and international investigations**

**Figure 3:**
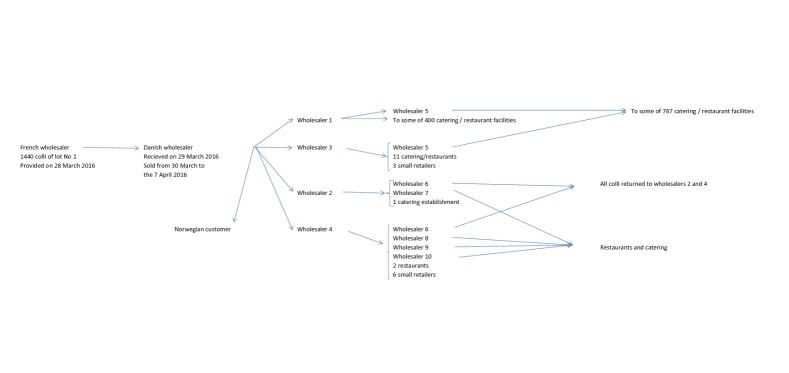
**Figure 3.** Trace-forward diagram describing the distribution chain of Lot 1 of the imported green coral lettuce related to a series of outbreaks of norovirus infections, Denmark, April 2016. One colli of lettuce consists of 8-12 heads. The heads were packaged individually by the Danish wholesaler.

Green coral lettuce of the *Lollo Bionda* variety was found to have been served in all outbreaks. The lettuce used or most probably used in the dishes served at the settings from where illness was reported was traced back through the distribution chain to two main lots (Lot 1 and Lot 2) packaged at a Danish establishment. The two lots were imported from France. Based on the distribution patterns, Lot 1 appeared as the most likely source of the outbreaks. When preliminary trace-back results became available, on 8 April, the wholesaler chose to withdraw this lot from the market on a voluntary basis. At that time, the possible involvement of Lot 2 was not fully known. A trace-forward analysis of this lot was then performed. Figure 3 illustrates the distribution chain of Lot 1. Part of the lot was sold on to Norway. However, this did as far as known not give rise to outbreaks in Norway (Dr. Line Vold, Norwegian Institute of Public Health, personal communication). Information about the possibility of norovirus outbreaks related to green lettuce was communicated to other EU countries via the RASSF system on 18 April (RASFF notification 2016.0468) and via the ECDC EPIS-FWD network on 20 April. No other EU countries reported to have experienced norovirus outbreaks with possible relation to this incident.

Information from the Danish packer and provided through the RASFF system showed that both Lots 1 and 2 were produced in France but by growers in two different regions. According to the registrations, both lots were packaged in succession on the same line on the same days and distributed at the same time. In response to the RASFF, The French Food Authorities reported that Lot 1 had been delivered only to Denmark and that norovirus was not detected at a follow-up control inspection at the site of the producer. Personnel handling the lettuce at the Danish packer had not reported illness in the relevant time period and no breaches in the hygiene procedures could be detected. However, it cannot be ruled out that cross contamination could have taken place in the packaging process. When this information became available, the shelf life of lettuce from Lot 2 had already been exceeded and was therefore not withdrawn by the DVFA.

**Microbiological investigations**

A total of 56 fecal samples from 31 patients were submitted for testing. Norovirus was detected in 50 of the samples from 28 patients comprising nine outbreaks. No other pathogenic viruses or bacteria were found. All positive norovirus results were of GI. Sequencing of both genetic regions (polymerase and capsid) was successfully performed on material from 17 patients. All but one were found to be genotype GI.P2-GI.2. Material from four patients only displayed sequences from the capsid area whereas polymerase alone was successfully sequenced from three patients. All of these were genotyped to GI.2 and GI.P2 respectively. In four cases, genotyping was unsuccessful. Upon alignment, sequences from all patients of either GI.P2 or GI.2 displayed 100% homology. The single patient with a different type was found to be infected with GI.Pb-GI.6. This patient was a part of outbreak 2 (Table 1), where samples from two other patients were positive for the outbreak genotype.

From eight sampling sites, a total of 20 heads of lettuce were collected and analysed for the presence of norovirus. A low level of norovirus GI (< tLOD and tLOQ but estimated to 8 copies per 25 g) was detected in an undiluted RNA extract of one sample of lettuce from Lot 2. Sequence analysis of a 223 bp fragment from the capsid region showed more than 99% homology (221/223 bp) with the sequences obtained from patients.

## DISCUSSION

Contaminated green coral lettuce produced in France was found to be the source of all of 23 separate point-source outbreaks of gastroenteritis occurring in Denmark during one week in April 2016. This evidence is supported by the consistency between exposure and illness, the unusually high number of individual outbreaks tightly clustered in time, the trace-back information, the results of the analytical studies, and of the virological analyses. Identical norovirus strains were detected in samples from patients in eight separate outbreaks, clearly linking them as part of the same overall meta-outbreak. Due to the symptoms and the relatedness of the outbreaks, it was concluded that norovirus was the cause of all the outbreaks. This illustrates the strength of using detailed subtyping of viral RNA from patient samples to link different outbreaks – a method we have previously used successfully on a series of norovirus outbreaks caused by frozen raspberries, and where the link between these outbreaks was far less obvious than here.^4^ Presence of norovirus in the lettuce was also confirmed by direct testing. This procedure is difficult, and was only successful for a single lettuce head following modifications of the PCR detection protocol (which is therefore listed in the Methods section). The lettuce head that tested positive was of Lot 2, indicating that this lot could also have contributed to the illnesses.

These outbreaks show that norovirus have the potential to affect many people in a short period of time if it appears in fresh produce. Fresh produce is increasingly being reported as the course of foodborne outbreaks of many etiologies, including noroviruses, and such outbreaks may occur more often than reported, especially when food is distributed to many different locations.^20^^,^ 21 Interestingly, Denmark experienced an almost identical incident of outbreaks in February 2010^22^^,^ 23 when imported green coral lettuce from France caused 21 separate, but linked outbreaks over the course of a few days. In 2010, however, the lettuce was additionally contaminated with enterotoxigenic* E. coli*.

Our investigations could not establish at which point in the production or packaging the contamination of lettuce occurred, but our investigation adds to the picture of leafy vegetables being a potential risk food for norovirus infections. The study highlights that trace-back investigation should be an integral part of epidemiological outbreak investigation and that maintaining product traceability is essential.

## Competing Interest Statement

The authors have declared that no competing interests exist.

## Corresponding Author

Luise Müller: lum@ssi.dk

## Data Availability Statement

Data is available upon request. Please contact Luise Müller: lum@ssi.dk.
